# The 111 Study: A Single-arm, Phase 3 Trial Evaluating One Cycle of Bleomycin, Etoposide, and Cisplatin as Adjuvant Chemotherapy in High-risk, Stage 1 Nonseminomatous or Combined Germ Cell Tumours of the Testis^☆^

**DOI:** 10.1016/j.eururo.2019.11.022

**Published:** 2020-03

**Authors:** Michael Cullen, Robert Huddart, Johnathan Joffe, Deborah Gardiner, Lauren Maynard, Paul Hutton, Danish Mazhar, Jonathan Shamash, Matthew Wheater, Jeff White, Aicha Goubar, Nuria Porta, Stephanie Witts, Rebecca Lewis, Emma Hall, on behalf of the 111 Trial Management Group

**Affiliations:** a University Hospitals Birmingham NHS Foundation Trust, Birmingham, UK; b The Institute Of Cancer Research, London, UK; c The Royal Marsden NHS Foundation Trust, London, UK; d The Leeds Teaching Hospitals NHS Trust, Leeds, UK; e Clinical Trials and Statistics Unit, The Institute of Cancer Research, London, UK; f Cambridge University Hospitals NHS Foundation Trust, Cambridge, UK; g Barts Health NHS Trust, London, UK; h University Hospital Southampton NHS Foundation Trust, Southampton, UK; i Beatson West of Scotland Cancer Centre, NHS Greater Glasgow and Clyde, Glasgow, UK

**Keywords:** Testicular cancer, NSGCTT, Surveillance, Chemotherapy, BEP, Phase III trial, Adjuvant therapy

## Abstract

**Background:**

Standard management in the UK for high-risk stage 1 nonseminoma germ cell tumours of the testis (NSGCTT) is two cycles of adjuvant bleomycin, etoposide (360 mg/m^2^), and cisplatin (BE_360_P) chemotherapy, or surveillance.

**Objective:**

To test whether one cycle of BE_500_P achieves similar recurrence rates to two cycles of BE_360_P.

**Design, setting, and participants:**

A total of 246 patients with vascular invasion–positive stage 1 NSGCTT or combined seminoma + NSGCTT were centrally registered in a single-arm prospective study.

**Intervention:**

One cycle comprising bleomycin 30000 IU on days 1, 8, and 15, etoposide 165 mg/m^2^ on days 1–3, and cisplatin 50 mg/m^2^ on days 1–2, plus antibacterial and granulocyte colony stimulating factor prophylaxis.

**Outcome measurements and statistical analysis:**

The primary endpoint was 2-yr malignant recurrence (MR); the aim was to exclude a rate of ≥5%. Participants had regular imaging and tumour marker (TM) assessment for 5 yr.

**Results and limitations:**

The median follow-up was 49 mo (interquartile range 37–60). Ten patients with rising TMs at baseline were excluded. Four patients had MR at 6, 7, 13, and 27 mo; all received second-line chemotherapy and surgery and three remained recurrence-free at 5 yr. The 2-yr MR rate was 1.3% (95% confidence interval 0.3–3.7%). Three patients developed nonmalignant recurrences with localised teratoma differentiated, rendered disease-free after surgery. Grade 3–4 febrile neutropenia occurred in 6.8% of participants.

**Conclusions:**

BE_500_P is safe and the 2-yr MR rate is consistent with that seen following two BE_360_P cycles. The 111 study is the largest prospective trial investigating one cycle of adjuvant BE_500_P in high-risk stage 1 NSGCTT. Adoption of one cycle of BE_500_P as standard would reduce overall exposure to chemotherapy in this young population.

**Patient summary:**

Removing the testicle fails to cure many patients with high-risk primary testicular cancer since undetectable cancers are often present elsewhere. A standard additional treatment in Europe is two cycles of chemotherapy to eradicate these. This trial shows one cycle has few adverse effects and comparable outcomes to those seen with two cycles.

## Take Home Message

The 111 trial aimed to exclude a 2-yr recurrence rate of >5% with adjuvant one cycle of bleomycin, etoposide (500 mg/m2), and cisplatin (BE500Px1) in stage 1, high-risk nonseminomatous germ cell testis tumours. This was achieved, with 1.3% malignant recurrences among 236 cases. BE500Px1 should replace BE360Px2 as standard care for such patients.

## 1 Introduction

Testicular cancer is the most common cancer among young men in Western populations and most patients present with stage 1 disease. Many nonseminomas and combined germ cell tumours of the testis (NSCGCTT) have vascular invasion (VI^+^) by malignant cells and are at high risk (˜50%) of harbouring undetected metastases [1], [2], confirmed consistently in many studies of surveillance [3].

Standard post-orchidectomy management options in Europe for this patient population are adjuvant chemotherapy (AC) with two cycles of bleomycin, etoposide, cisplatin (BE_360_Px2) or surveillance with BE_500_Px3 on recurrence [4]. Adjuvant BE_360_Px2 results in malignant recurrence rates of <5%. Both management options yield cure rates approaching 100% [5], [6]. According to proponents of surveillance, 50% of patients receive unnecessary AC [7], while AC proponents highlight poor adherence to surveillance and recurrence with advanced disease sometimes requiring retroperitoneal lymph node dissection (RPLND) [8]. It is clearly important to expose patients to the minimum treatment necessary. The frequency of immediate and late chemotherapy toxicity is closely related to total doses received; if AC BE_500_Px1 were as effective as BE_360_Px2, the former would substantially reduce the total chemotherapy burden since approximately half of surveillance cases recur, requiring BE_500_Px3.

Over recent years evidence has accumulated supporting the efficacy of BE_500_Px1 [9], [10], [11], [12], [13]; nevertheless, uptake of single-cycle AC remains patchy.

The 111 study was designed as a practice-changing trial to confirm the efficacy signals from these smaller studies. It tested BE_500_Px1 in a prospective, multicentre, single-arm trial in a patient population with an expected risk of recurrence of ˜50%. On the basis of the experience of key opinion leaders and trial collaborators in testicular cancer and existing data, the figure considered acceptable for relapse after BE_500_Px1 was <5%. The aim was to demonstrate whether AC with BE_500_Px1 confers a 2-yr malignant recurrence (MR) rate <5% in high-risk stage one testicular NSCGCT, with acceptable short-term toxicity in line with, and no worse than, the established toxicity profile for patients receiving BE_360_Px2.

## 2 Patients and methods

### 2.1 Study design and participants

BEP111 is a single-group, nonrandomised, open-label, multicentre phase 3 trial of novel design using sequential application of defined stopping rules based on robust historical MR rate data for BE_360_Px2 and monitored by an independent data monitoring committee (IDMC). The trial, conducted in accordance with the principles of good clinical practice, was approved by the Medicines and Healthcare Products Regulatory Authority and London (South East) Research Ethics Committee (09/H1102/86) and co-sponsored by University Hospitals Birmingham NHS Trust and The Institute of Cancer Research (ICR). The study is registered (ISRCTN37875250). All participants provided written informed consent. The Clinical Trials and Statistics Unit at the ICR (ICR-CTSU) coordinated the study and carried out central data management, statistical data monitoring, and all analyses. The trial was overseen by an independent trial steering committee.

Patients newly diagnosed with VI^+^ stage 1 NSCGCTT who were able to start chemotherapy ideally within 6 wk of orchidectomy (but no later than 8 wk unless agreed by the chief investigator with a repeat CT scan to confirm stage 1) were eligible; Table 1 lists the full eligibility criteria. Baseline assessments included CT of the chest, abdomen, and pelvis and measurement of TMs (α-foetoprotein [AFP], lactate dehydrogenase, and human chorionic gonadotropin [HCG]) to confirm stage 1 disease. Patients were centrally registered with ICR-CTSU before commencing treatment.

**Table 1:** Eligibility criteria for entry into the 111 trial.

Inclusion criteria	Exclusion criteria
Newly diagnosed, histologically proven pure NSGCT or combined seminoma + NSGCT of the testis	Previous chemotherapy
Vascular invasion of primary tumour into testicular veins or lymphatics	Previous malignant disease
Stage 1B (T2N0M0), evidence of no metastases on CT or tumour marker (AFP, HCG) estimations a	Liver function impairment (bilirubin >1.25 × upper limit of normal for reporting laboratory)
Age ≥16 yr	Pre-existing neuropathy
Fit to receive chemotherapy	Pulmonary fibrosis
Creatinine clearance >50 ml/min	Serious illness or medical conditions incompatible with safe protocol treatment
WBCs >1.5 × 10^9^ /l and platelets >100 × 10^9^ /l	
Able to start BEP chemotherapy within 6 wk of orchidectomy	
Written informed consent	

NSGCT = nonseminomatous germ cell tumour; AFP = α-foetoprotein; HCG = human chorionic gonadotropin; WBCs = white blood cells; BEP = bleomycin, etoposide, and cisplatin.

a In cases in which markers were raised before orchidectomy, an optimum marker decline approaching normal levels was required postoperatively before commencing trial therapy.

### 2.2 Procedures

Participants received BE_500_Px1 over 3 wk (bleomycin 30 000 IU on days 1, 8, and 15, cisplatin 50 mg/m^2^ on days 1–2, etoposide 165 mg/m^2^ on days 1–3). Prophylaxis with an oral fluoroquinolone antibacterial [14] and subcutaneous granulocyte colony stimulating factor (GCSF) was mandated to reduce neutropenic sepsis [15].

Patients had a full clinical assessment including grading of adverse events (AEs) using the National Cancer Institute’s Common Toxicity Criteria for Adverse Events (CTCAE v3) no later than 4 wk following BE_500_Px1, then every 2 mo until 6 mo, every 3 mo until 24 mo, every 4 mo during the third year, and every 6 mo during the fourth and fifth years after treatment. Computed tomography (CT) scans of the chest, abdomen, and pelvis were required at 6, 12, 24, and 60 mo, with a chest X-ray at all other visits. A physical examination and TM measurements were required at each visit to assess signs of recurrence or development of a second primary tumour.

### 2.3 Outcomes

For analysis purposes, recurrences were defined using two categories. MR was defined as a recurrence indicated by rising TM (AFP and/or HCG) from two consecutive results taken ≥1 wk apart showing a >50% increase above the upper limit of normal and/or a histologically MR (eg, undifferentiated, yolk sac, or choriocarcinoma) and/or recurrence at multiple sites. Benign recurrence (BR) was defined as a single-site recurrence with no TM elevation, consisting of fully resected, differentiated teratoma (TD) with no histological evidence of viable malignancy. This does not imply failure of AC, since TD is unresponsive to chemotherapy and is analogous to “growing teratoma” syndrome after chemotherapy for metastatic disease. All recurrences were prospectively reviewed and classified by the chief investigator and the IDMC.

The primary endpoint was the MR rate at 2 yr. Secondary efficacy outcome measures included the BR rate, overall recurrence rate, development of contralateral second primary testicular germ cell malignancy, relapse-free survival (defined as the time from registration until first confirmed relapse or death from any cause), and overall survival. Additional secondary endpoints were immediate and delayed toxicity. Treatment-emergent acute toxicity was any AE not present before initiation of the trial treatment or already present but worsening following exposure to the trial treatment. Delayed toxicity was reported for the time intervals 2–12 mo, 18–24 mo, and >24 mo. Emergent delayed toxicity within 2–12 mo was any AE that was not present or worsened from baseline or end of cycle.

### 2.4 Statistical analysis

The trial was powered to exclude a 2-year MR rate ≥5% in high-risk stage 1 NSCGCTT. Based on exact binomial probabilities with 80% power and a one-sided α of 5%, the minimum sample size required was 236 patients. In practice this means that if ≥230 patients remained MR-free, the true MR rate is highly likely to be <5%.

After each recurrence event, sequential early stopping rules for futility were applied based on the probability of the final relapse rate being ≥5% (conditional on the data and follow-up available at that time), as monitored by the IDMC. Adequate β spending functions were chosen via simulation to ensure that despite multiple analyses the final α and power are 5% and 80%, respectively. A formal interim analysis was conducted when 157 patients had been followed up for ≥2 yr.

Analyses of outcomes included all eligible registered patients. For safety endpoints, analyses were according to treatment received. The MR rate at 2 yr and its 95% confidence interval (CI) were estimated using exact binomial probabilities. Patients without complete data at 2 yr of follow-up were assumed to have no MR at 2 yr. To account for such censoring, the 2-yr MR rate was also estimated using the Kaplan-Meier method. Patients with BR were censored at the time of the event. Both methods had to yield upper 95% CI limits of <5% to exclude an MR rate ≥5%. Sensitivity analyses of the primary endpoint were performed for the per protocol population.

Similar analysis methods were used for other efficacy endpoints. In the absence of a discrepancy between the exact binomial and Kaplan-Meier methods, the latter are reported. The frequency and nature of toxicities are summarised using the worst CTCAE grade for each of the reporting periods (end of cycle, delayed 2–12, 18–24, and >24 mo). Analyses were based on a database snapshot taken December 4, 2017 and were performed using Stata v13.1 [16].

## 3 Results

**Fig. 1:**
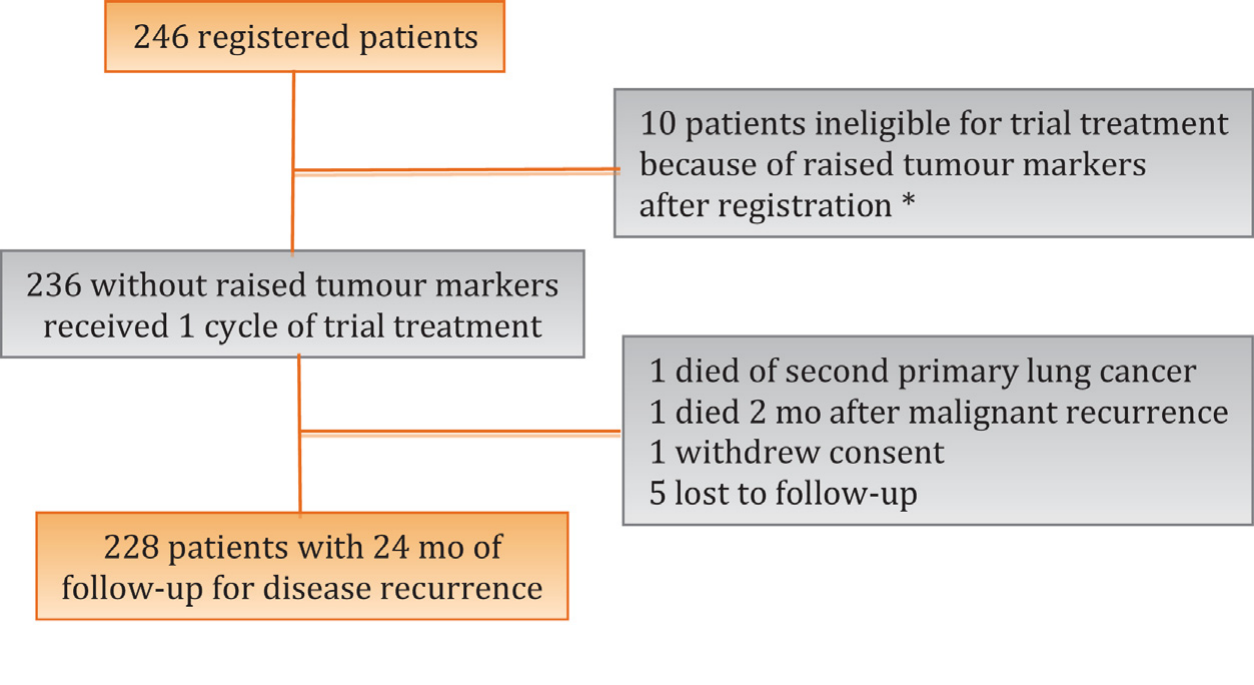
CONSORT diagram. * Ineligibility confirmed by central review. Patients followed-up but data are not included within the primary intention-to-treat analysis in accordance with the statistical analysis plan.

Between February 18, 2010 and March 31, 2014, 246 patients were registered from 33 UK NHS hospitals (Fig. 1), all of which were peer-reviewed accredited testis tumour treatment centres. The median follow-up at the time of reporting is 49 mo (interquartile range [IQR] 37–60). Ten patients were replaced after they were identified as ineligible following registration because of rising TMs. In 114/246 cases (46%) there was histopathological evidence of seminoma in addition to unequivocal VI^+^ NSGCTT (Table 2). Of the 236 patients included in the analysis, 228 (97%) were followed up to at least 2 yr.

**Table 2:** Patient characteristics on entry into the 111 trial.

		
Age (*n = 246*) median 31 IQR (25,39)	≤24 yr	57 (23)
	25–29 yr	65 (26)
	30–39 yr	70 (29)
	40–49 yr	38 (15)
	≥50 yr	16 (6.5)
WHO performance status (*n = 239*)	0	230 (96)
	1	9 (3.8)
Tumour diameter (cm) (*n = 239*)	<2	47 (20)
	2-5	121 (51)
	>5	71 (30)
Histopathology type (*n = 246*)	Pure NSGCTT	132 (54)
	Combined seminoma + NSGCTT	114 (46)
Pathological tumour stage (*n = 246*)	pT2 (blood vessel and or lymphatic invasion, VI^+^)	237 (96)
	pT3 (VI^+^ and tumour extending to the spermatic cord)	9 (3.7)

NSGCTT = nonseminoma germ cell tumour of the testis.

The median time between orchidectomy and the start of treatment was 6 wk (IQR 5–7) and all 236 patients started BE_500_P. Treatment was received as planned by 221/236 (94%) of eligible patients. Eight patients (3.4%) received a per-protocol bleomycin dose reduction because of neutropenia. There was good adherence to neutropenic sepsis prophylaxis, with 219/236 (93%) receiving this per protocol. The remaining 17 patients received some prophylaxis (either GCSF or antibacterial).

There were four MR cases at 6, 7, 13, and 27 mo after trial registration, all of which were confirmed as malignant NSGCT via histological examination and/or rising TMs (Table 3). The 2-yr MR rate is 1.3% estimated using exact binomial probabilities (95% CI 0.3–3.7%) and Kaplan-Meier methods (95% CI 0.4–4.0%). With both methods, a 2-yr MR rate ≥5% can be excluded. The 4-yr MR rate is 1.8% (95% CI 0.7–4.6%). All four MR cases required surgical intervention and second-line chemotherapy. Three patients achieved complete remission, remaining well 5 yr after treatment. The patient with MR at 6 mo had very extensive, unresectable retroperitoneal NSGCT that failed to respond to chemotherapy, and the patient died 2 mo later. This was the only case of MR with an International Germ Cell Cancer Collaborative Group metastatic prognostic classification of intermediate; all others fell in the good prognosis category [17].

**Table 3:** Details of all recurrences in the population analysed (*n* = 236).

	Age at BL (yr)	Histology type (orchidectomy)	TS (cm)	TRR (mo)	Site of recurrence	IGCCCG prognostic category		Surgical management	Chemotherapy regimen and cycles	Outcome (last FU, mo)
Malignant										
1	55	Pure NSGCTT	>5	5.8	RPLN + raised AFP	Intermediate (LDH 1.5–10 × ULN)	Attempted RPLND. Extensive unresectable tumours		IPE × 2	Died at 9 mo with resistant malignant NSGCT
2	24	Pure NSGCTT	>5	6.7	Lung	Good	Video assisted wedge resection		TIP × 4	CR (60.9)
3	42	Combined seminoma + NSGCTT	2–5	12.5	RPLN + raised AFP	Good	RPLND		BEP × 3	CR (60.4)
4	31	Combined seminoma + NSGCTT	2–5	27.1	Right inguinal region + raised HCG	Good	Excision of spermatic cord and external iliac lymph node		TIP × 3	CR (62.6)
Benign										
1	22	Pure NSGCTT	2–5	6.8	RPLN	Good	RPLND		None	CR (61.9)
2	22	Combined seminoma + NSGCTT	2–5	10.2	RPLN	Good	RPLND		None	CR (36.2)
3	29	Pure NSGCTT	<2	13.1	RPLN	Good	RPLND		None	CR (37.3)

BL = baseline; TRR = time of recurrence from registration; TS = tumour size at orchidectomy; IGCCCG = International Germ Cell Cancer Collaborative Group; RPLN = retroperitoneal lymph node; RPLND = RPLN dissection; IPE = ifosfamide, cisplatin, and etoposide; NSGCTT = nonseminoma germ cell tumour; FU = follow-up; CR = complete remission; TIP = paclitaxel, ifosfamide, and cisplatin; BEP = bleomycin, etoposide, and cisplatin; LDH = lactate dehydrogenase; ULN = upper limit of normal.

There were three BR cases consisting exclusively of histologically confirmed TD with no evidence of viable cancer at 7, 10, and 13 mo after trial registration (Table 3). All three underwent RPLND and remained well at 55, 26, and 24 mo following BR.

**Fig. 2:**
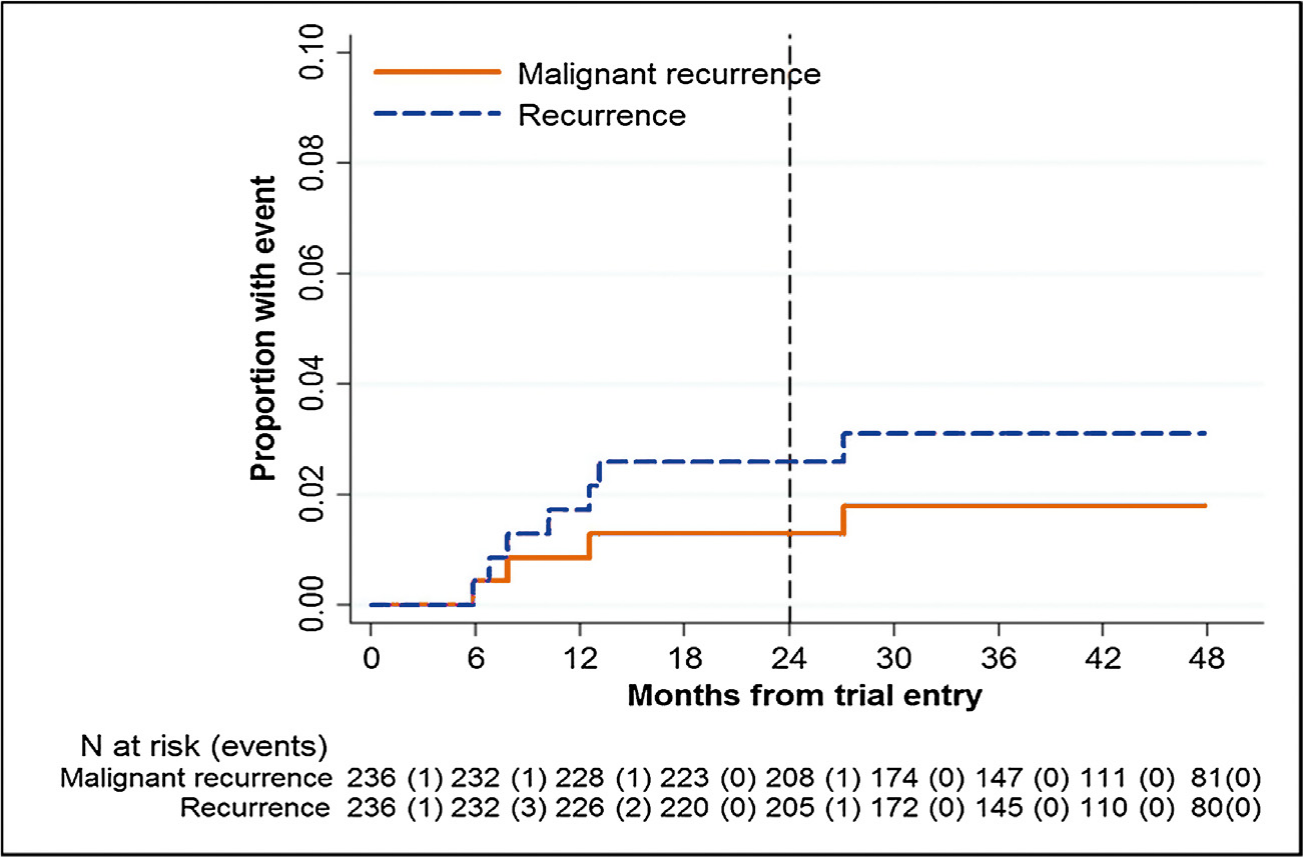
Recurrence rate estimated using the Kaplan-Meier method.

The MR + BR rate is 2.6% (95% CI 1.2–5.7%) at 2 yr and 3.1% (95% CI 1.5–6.3%) at 4 yr (Fig. 2).

Sensitivity analysis for the per protocol population (consisting of 208 eligible patients, compliant with treatment and with complete 2-yr follow-up) provided a 2-yr MR rate of 1.5% (95% CI 0.5–4.4%), while the MR + BR rate at 2 yr was 2.4% (95% CI 1.0–5.7%).

No cases of contralateral second primary testicular germ cell malignancy were reported. The 2-yr relapse-free survival was 97% (95% CI 94–99%). There were two unrelated deaths in patients free from recurrent testicular cancer: one due to small cell lung cancer at 18 mo after trial registration, and one from a self-administered drug overdose at 45 mo. The 2-yr overall survival is 99% (95% CI 97–100%).

Acute emergent toxicity within 4 wk following BE_500_P was assessed for 233/236 cases with paired baseline and end-of-cycle assessments. Ninety-five patients (41%) had at least one severe (grade 3–4) toxicity, including: neutropenia, 75 (32%); leukopenia, 40 (17%); febrile neutropenia (FN), 16 (6.8%); thrombocytopenia, 8 (3.4%); non-neutropenic sepsis, 7 (3.0%); and emesis, 6 (2.6%). Fewer than 3% of patients reported grade 3–4 late emergent toxicities (Table 4). Data on fertility indices will be published separately.

**Table 4:** Delayed toxicity: adverse event of worst CTCAE grade per patient^a^.

	Patients, *n* (%)					
	2–12 mo (*n* = 233) b		18–24 mo (*n* = 215)		>24 mo (*n* = 184)	
	Grade 1+	Grade 3+	Grade 1+	Grade 3+	Grade 1+	Grade 3+
Any toxicity	137 (59)	6 (2.6)	107 (50)	2 (0.9)	79 (43)	3 (1.6)
Specific toxicities of interest						
Dyspnoea	15 (6.4)	0 (0.0)	10 (4.7)	0	8 (4.3)	0
Ear and labyrinth disorders c	17 (7.3)	2 (0.9)	17 (7.9)	1 (0.5)	7 (3.8)	1 (0.5)
Psychiatric disorders d	9 (3.9)	1 (0.4)	3 (1.4)	0	4 (2.2)	0
Fatigue	4 (1.7)	0	0	0	1 (0.5)	0
Insomnia	2 (0.9)	0	2 (0.9)	0	1 (0.5)	0

CTCAE = Common Toxicity Criteria for Adverse Events.

a Details of grade 3–4 toxicities: 2–12 mo: grade 3 anaemia, ototoxicity (*n* = 2), weight increase, and depression, grade 4 thrombocytopenia and osteonecrosis; 18–24 mo: grade 3 osteonecrosis, ototoxicity, and tinnitus; >24 mo: grade3 diabetes and lethargy, grade 4 deafness.

b For the reporting period of 2–12 mo, emergent toxicities are presented (not present at or worsening from baseline or end of cycle). For the other reporting periods, toxicities were as reported.

c Ototoxicity, deafness, or tinnitus.

d Includes depression, anxiety, depressed mood, and altered mood.

## 4 Discussion

The 111 trial has demonstrated the efficacy of adjuvant BE_500_Px1 for high-risk (VI+) stage 1 NSCGCTT. The 2- and 4-yr MR rates of just 1.3% and 1.8%, respectively, are almost identical to the results reported following BE_360_Px2 [5], [10], [18], [19]. As seen in other studies of AC in this patient group [20], an additional three patients developed localised BR due, we believe, to growing teratoma resulting from successful treatment of malignant disease. The pragmatic decision to rely on a nonrandomised trial design was made in light of the rarity of the patient group under study and the low expected event rate in the study population. A noninferiority trial to demonstrate that one cycle was no worse than 3% less effective than two cycles (80% power, one-sided α of 5%) would have required 1110 participants, an impossible target within a reasonable timeframe.

The 111 trial design was developed in collaboration with investigators to identify an acceptable MR rate with BE_500_Px1 that would lead to adoption of the regimen, and thus fulfilled phase 3 criteria. This design was cited as a model option in a recent review of novel research methods aiming to change clinical practice for patients with rare cancers [21].

The MR rates observed in the 111 trial are consistent with three small, single-centre studies involving 112 patients [11], [12], [13]. They also reflect findings in a population-based study by the Swedish and Norwegian Testicular Cancer Project that included patients with low or high risk treated with BE_500_Px1 or BE_500_Px2. In their latest update [22], among 258 VI^+^ patients who chose BE_500_Px1 there were eight cases of MR (3.2%; 95% CI 1.6–6.4%) during median follow-up of 7.9 yr. A randomised German trial of BE_500_Px1 versus RPLND reported only two recurrences among 191 patients randomised to BE_500_Px1 (only one of which was malignant), but just 42% of randomised cases were classified as high risk and the outcome for this subgroup was not reported separately [9]. The authors concluded that their data “should encourage investigators to test the promising approach of one course BE_500_P”.

FN remains a serious risk with full-dose etoposide chemotherapy, with occasional fatalities, which is why we used dual infection prophylaxis in this adjuvant context. This appears to have been effective, since the rate of severe FN was 6.8% (with no deaths), compared to 20% following cycle 1 among 111 control testicular cancer patients receiving BEP and allocated to placebo in a randomised trial of prophylactic levofloxacin [15].

Late toxicity is a clear concern with adjuvant BE_500_P. A small number of patients (<3%) developed grade 3–4 late toxicity. There is ample evidence in testicular cancer of a direct relationship between cycle number (ie, cumulative dose) and delayed toxicity in terms of infertility, metabolic syndrome, neuropathy, and lung and renal function [23], [24], [25], [26], [27]. However, any toxicity developing after BE_500_Px1 has to be balanced against the greater risk of toxicity with the higher doses that would be given to the 50% of patients expected to relapse on a surveillance programme. Post-treatment fertility indices will be reported separately, but on the basis of published data following BE_360_Px2 it is unlikely that serious impairment of spermatogenesis will be demonstrated following one cycle [23].

The German and Scandinavian studies cited provided important foundations and a rationale for the present trial [9], [10]. Since their publication there has been controversy surrounding the options of AC versus surveillance in stage 1 NSGCTT. In their 2013 paper, Nichols et al [7] clearly favour surveillance. However, important differences between testicular cancer types and risk categories are obfuscated in this review. For instance, the authors mention recent trends towards less intensive surveillance with fewer CT scans and hence less radiation exposure. However, two studies cited in support excluded high-risk stage 1 NSGCTT [28], [29]. The authors also failed to consider the risk of requiring elective surgery (commonly RPLND) following chemotherapy for recurrence on surveillance. de Wit [8] noted that in the largest recent study of surveillance, 26% of relapsing patients required post-chemotherapy surgery [6]. In the 111 trial, 3% of patients (7/236) required surgery for MR or BR. The much higher level of surgery required among surveillance patients relates to more advanced disease stages at the time of chemotherapy exposure. This drawback is exacerbated by poor compliance with surveillance schedules, as reported in several studies, particularly in those relating to surveillance in the community setting [30]. Treatment of MR, although usually successful, involves more intensive chemotherapy and major surgery and is extremely disruptive to the lives of young men and their families. RPLND has been used in this scenario as an alternative, but a German study showed that recurrences were more frequent among unselected stage pN0 NSGCT patients than after adjuvant BEP chemotherapy (8% vs 1%), and in VI^+^ patients the recurrence rate is 28% [31] unless adjuvant chemotherapy is used in pN + cases.

## 5 Conclusions

The 111 study is the first prospective trial of BE_500_Px1 with sufficient high-risk stage 1 NSGCTT or combined seminoma + NSGCTT patients to exclude an MR rate at 2 yr of ≥5%. Despite the unavoidable limitation of being a single-arm study, 111 achieved its aim, with a malignant failure rate of just 1.3% and very low levels of serious short-term and delayed toxicity. This trial confirms that BE_500_Px1 should replace BE_360_Px2 as the standard adjuvant therapy offered to all patients with VI^+^ stage 1 NSCGCTT.

  ***Author contributions***: Robert Huddart had full access to all the data in the study and takes responsibility for the integrity of the data and the accuracy of the data analysis.

*Study concept and design*: Cullen, Huddart, Joffe, Hall.

*Acquisition of data*: Gardiner, Lewis, Witts.

*Analysis and interpretation of data*: Cullen, Huddart, Joffe, Hall, Porta, Goubar, Maynard.

*Drafting of the manuscript*: Cullen, Huddart, Joffe, Hall, Lewis, Gardiner, Porta, Goubar, Witts, Maynard, Hutton, Mazhar, Shamash, Wheater, White.

*Critical revision of the manuscript for important intellectual content*: Cullen, Huddart, Joffe, Hall, Lewis, Gardiner, Porta.

*Statistical analysis*: Hall, Porta, Goubar, Maynard.

*Obtaining funding*: Cullen, Huddart, Joffe, Hall.

*Administrative, technical, or material support*: Gardiner, Lewis, Witts.

*Supervision*: Cullen, Huddart, Joffe, Hall.

*Other*: None.

  ***Financial disclosures:*** Robert Huddart certifies that all conflicts of interest, including specific financial interests and relationships and affiliations relevant to the subject matter or materials discussed in the manuscript (eg, employment/affiliation, grants or funding, consultancies, honoraria, stock ownership or options, expert testimony, royalties, or patents filed, received, or pending), are the following: Emma Hall reports grants from Cancer Research UK during the conduct of the study; and grants from Merck Sharp & Dohme, Janssen-Cilag, Aventis Pharma (Sanofi), and Accuray Inc., and grants and nonfinancial support from AstraZeneca and Bayer outside the submitted work. Robert Huddart reports nonfinancial support from Janssen, grants and personal fees from MSD, personal fees and nonfinancial support from Roche, personal fees from Bristol Myers Squibb, and grants from Cancer Research UK outside the submitted work. The remaining authors have nothing to disclose.

  ***Funding/Support and role of the sponsor*:** 111 is co-sponsored by The Institute of Cancer Research and University Hospitals Birmingham NHS Foundation Trust. The study was funded by Cancer Research UK (CRUK/09/011) and Queen Elizabeth Hospital Birmingham Charity. The funders of the study had no role in study design, data collection, data analysis, data interpretation, or writing of the report. ICR-CTSU receives programme grant funding from Cancer Research UK (grant C1491/A15955). Support was also received from the National Institute for Health Research (NIHR) Cancer Research Network (CRN). We acknowledge NHS funding to the NIHR Biomedical Research Centre at The Royal Marsden and the ICR.

## Appendix A Recruiting consultants according to centre (number of patients recruited are shown in bold font)

Queen Elizabeth Hospital, Birmingham, **23**, Prof. M. Cullen, Dr. E. Porfiri; Royal Marsden Hospital, Sutton, **23**, Prof. R. Huddart; Beatson West of Scotland Cancer Centre, Glasgow, **21**, Dr. J. White, Dr. A. Waterston; St. James’s University Hospital, Leeds, **21**, Dr. J. Joffe, Dr. D. Stark; Bristol Haematology and Oncology Centre, **16**, Dr. J. Braybrook; St. Bartholomew’s Hospital, London, **14**, Dr. J. Shamash; Southampton General Hospital, **13**, Dr. M. Wheater, Dr. P. Simmonds, Dr. G. Mead; Addenbrooke’s Hospital, Cambridge, **9**, Dr. D. Mazhar; Castle Hill Hospital, Hull, **9**, Dr. M. Butt; Royal Liverpool & Broadgreen University Hospital, **9**, Prof. P. Clark; Weston Park Hospital, Sheffield, **9**, Dr. L. Evans, Prof. R. Coleman; Leicester Royal Infirmary, **8**, Dr. G. Faust, Dr. C. Esler; Nottingham University Hospital, **8**, Dr. I. Hennig; Royal Sussex County Hospital, Brighton, **7**, Dr. D. Bloomfield; Clatterbridge Cancer Centre, Wirral, **6**, Prof. P. Clark, Dr. J. Carser; Royal Devon & Exeter Hospital, Exeter, **6**, Dr. A. Hong; University College Hospital, London, **6**, Dr. S. Harland; Western General Hospital, Edinburgh, **5**, Dr. A. Law, Dr. D. McLaren; Aberdeen Royal Infirmary, **4**, Dr. G. MacDonald; Guy’s Hospital, London, **4**, Dr. S. Rudman, Dr. S. Chowdhury; Maidstone Hospital, **4**, Dr. S. Beesley, Dr. H. Taylor; Royal Derby Hospital, **3**, Dr. P. Chakraborti; Royal Surrey County Hospital, Guildford, **3**, Dr. J. Money-Kyrle; Lincoln County Hospital, **2**, Dr. T. Sreenivasan; Norfolk & Norwich University Hosptial, **2**, Dr. G. Kapur, Dr. S. Alexander; Pilgrim Hospital, Boston, **2**, Dr. Addeo; Royal Berkshire Hospital, Reading, **2**, Dr. P. Rogers; Ysbyty Gwynedd Hospital, Bangor, **2**, Dr. N. Stuart; Cheltenham General Hospital, **1**, Dr. R. Owen; Churchill Hospital, Oxford, **1**, Dr. A. Protheroe; Ipswich Hospital, **1**, Dr. R. Venkitaraman; University Hospitals of Coventry & Warwickshire NHS Trust, **1**, Dr. A. Stockdale; Velindre Cancer Centre, Cardiff, **1**, Dr. S. Kumar.
